# Optimization of transplastomic production of hemicellulases in tobacco: effects of expression cassette configuration and tobacco cultivar used as production platform on recombinant protein yields

**DOI:** 10.1186/1754-6834-6-65

**Published:** 2013-05-03

**Authors:** Igor Kolotilin, Angelo Kaldis, Eridan Orlando Pereira, Serge Laberge, Rima Menassa

**Affiliations:** 1 Southern Crop Protection and Food Research Centre, Agriculture and Agri-Food Canada, London, ON, Canada; 2 Department of Biology, Western University, London, ON, Canada; 3 Soils and Crops Research Development Center, Agriculture and Agri-Food Canada, Québec, QC, Canada

**Keywords:** Transplastomic tobacco, Recombinant proteins, Cellulolytic enzymes, Chloroplast expression cassette, Cellulosic biofuels

## Abstract

**Background:**

Chloroplast transformation in tobacco has been used extensively to produce recombinant proteins and enzymes. Chloroplast expression cassettes can be designed with different configurations of the cis-acting elements that govern foreign gene expression. With the aim to optimize production of recombinant hemicellulases in transplastomic tobacco, we developed a set of cassettes that incorporate elements known to facilitate protein expression in chloroplasts and examined expression and accumulation of a bacterial xylanase XynA. Biomass production is another important factor in achieving sustainable and high-volume production of cellulolytic enzymes. Therefore, we compared productivity of two tobacco cultivars – a low-alkaloid and a high-biomass - as transplastomic expression platforms.

**Results:**

Four different cassettes expressing XynA produced various mutant phenotypes of the transplastomic plants, affected their growth rate and resulted in different accumulation levels of the XynA enzyme. The most productive cassette was identified and used further to express XynA and two additional fungal xylanases, Xyn10A and Xyn11B, in a high-biomass tobacco cultivar. The high biomass cultivar allowed for a 60% increase in XynA production per plant. Accumulation of the fungal enzymes reached more than 10-fold higher levels than the bacterial enzyme, constituting up to 6% of the total soluble protein in the leaf tissue. Use of a well-characterized translational enhancer with the selected expression cassette revealed inconsistent effects on accumulation of the recombinant xylanases. Additionally, differences in the enzymatic activity of crude plant extracts measured in leaves of different age suggest presence of a specific xylanase inhibitor in the green leaf tissue.

**Conclusion:**

Our results demonstrate the pivotal importance of the expression cassette design and appropriate tobacco cultivar for high-level transplastomic production of recombinant proteins.

## Background

Chloroplasts, the photosynthetic organelles in plant cells, are believed to originate from endosymbiotic cyanobacteria that were incorporated into an ancestral eukaryotic host cell [1]. Although the bulk of the endosymbionts' genome was depleted during evolution, chloroplasts retain a relatively small circular genome (plastome) that is highly polyploid, and the chloroplast genetic machinery for transcription/translation resembles that of prokaryotes [2,3]. These features make plastome transformation in higher plants an alternative to nuclear genome transformation, and also offer several advantages, such as 1) integration of the transgene into a precise plastome locus by homologous recombination; 2) lack of positional effects and epigenetic factors, such as transgene silencing, often detrimental for foreign protein expression in nuclear transformants; 3) the ability of the plastid genetic machinery to transcribe and translate operons and, 4) transgene containment due to maternal inheritance of the engineered plastome [4-6]; for review see [7-10]. Stable chloroplast genome transformation was achieved in several plant species, where routine generation of transplastomic plants with well-established protocols have been developed mostly in *Solanaceae* species such as tobacco, tomato and potato [4,11-16]

Since the development of the plastome transformation techniques more than two decades ago, successful production in chloroplasts of heterologous proteins from various origins has been reported [11,17-19]. Many chloroplast transformation vectors with different configuration of the chloroplast expression cassettes were designed and applied [7,11,16,17]. A typical cassette would contain a gene of interest (GOI) and a selectable marker - a gene for antibiotic resistance that enables selection of transplastomic clones, most commonly the aminoglycoside adenylyltransferase (*aadA*) gene conferring resistance to streptomycin/spectinomycin [14,20]. Expression of these genes is regulated by specific cis-acting elements that are usually adopted from endogenous as well as heterologous plastid genes and include chloroplast promoters, 5'- and 3'-untranslated (UTR) sequences and ribosome-binding sites [11,16,18]. Structural design (configuration) of a cassette can vary according to the plastome locus where integration is planned; usually transcriptionally-active or silent intergenic spacer as well as cassettes of different configuration can be introduced into the same plastome locus. Various cassettes, designed by different research groups, have been integrated into several distinct sites in the tobacco plastome, mostly targeting intergenic spacers in the inverted repeat (IR) region between the *trnV* and *rps12* genes, an intron with no read-through transcription, and between the *trnI* and *trnA* genes, a transcriptionally-active intron, where endogenous transcriptional activity may be exploited to express foreign genes. In the large single copy (LSC) region a silent intergenic spacer between *trnfM* and *trnG* genes has been also extensively utilized [6,11,16,21,22]. Cassettes integrated into these plastome loci were reported to produce abundant yields of recombinant proteins, some reaching a massive accumulation of 70% of the total soluble protein (TSP) in the plant leaf tissue and overburdening the protein synthesis machinery in the plastid [23-25]. Studies that addressed the reasons of variable levels of recombinant protein accumulation in chloroplasts showed that multiple determinants at transcriptional, translational and post-translational levels are involved in the process. Factors such as mRNA stability, mRNA-rRNA interactions, appropriate codon usage, efficient processing of polycistronic transcripts, the N-terminal amino acid residue and sequences downstream the initial methionine of the nascent polypeptide chain, as well as protein secondary structure - all exert tight control over recombinant protein production and accumulation in chloroplasts [26-30].

Although numerous different cassettes have been constructed by several laboratories and introduced into the tobacco plastome to express various proteins, the assortment of the cis-acting elements used to facilitate the expression of the genes of interest and the selectable marker genes from these cassettes remains limited. Typically, a strong chloroplast ribosomal operon promoter (P*rrn*) and the promoter for the PSII protein D1 (P*psbA*) are used, driving the transcription of the foreign genes [16,18]. In most constructs reported, the mRNA transcripts of the transgenes were stabilized by 5' and 3' UTRs of the tobacco endogenous plastid *psbA*, *rbcL* or *rps16* genes; heterologous UTRs originating from different species were also successfully implemented [31-34]. Coding sequences of the genes of interest and the selectable marker may be fused at the 5’ to translational enhancers, also called “downstream boxes” (DB) - specific DNA sequences, that have been previously identified as important regulators of translation efficiency and defined by the 10–15 codons immediately downstream of the initial ATG start codon [16,26,27,35-39]. One of the best-characterized in that context is the N-terminal portion of the protein encoded by gene 10 from bacteriophage T7 (T7*g10*), which has been shown to increase accumulation of several recombinant proteins produced in chloroplasts [32,40,41].

In an attempt to optimise production of cellulolytic enzymes in transplastomic tobacco, we used a set of endogenous and heterologous cis-acting elements to construct several cassettes with varying configuration of the cis-acting regulatory elements and the foreign genes expressed. We introduced four different cassettes containing the *aadA* gene and the *xynA* gene encoding a bacterial xylanase from *Clostridium cellulovorans* into the *trnI - trnA* intron of the tobacco plastome. We confirmed the functionality of the best cassette with two additional fungal xylanases, *Aspergillus niger* Xyn10A or Xyn11B using a different, high-biomass tobacco cultivar. Cumulatively, our results demonstrate the importance of two factors for optimization of transplastomic production of recombinant proteins in tobacco: 1) effective structural design of the cassette and 2) the choice of regulatory cis-elements. Developmental restriction of some transplastomic plants may be considered an additional limiting factor to obtainable yields of the recombinant protein.

## Results and discussion

### General considerations

**Figure 1:**
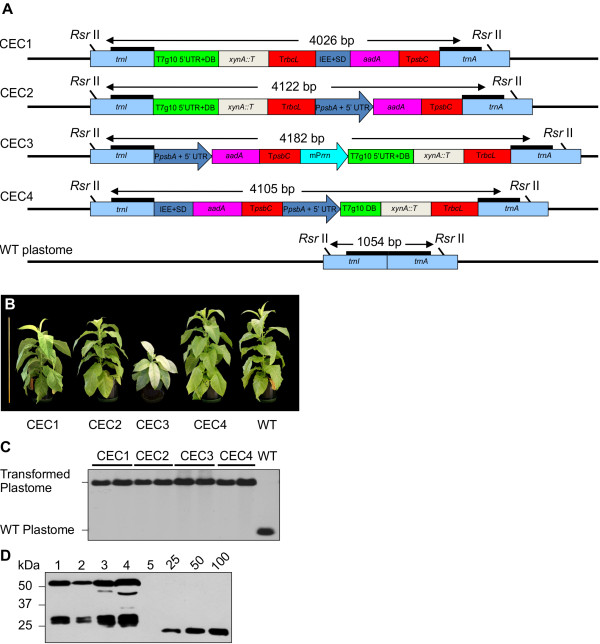
**Chloroplast Expression Cassettes (CECs) used in this study; Phenotype of primary transformants (cv. 81V9); Confirmation of homoplastomy; Confirmation of XynA expression. A**. Four Chloroplast Expression Cassettes (CECs, designated CEC1 through -C4) with varying configuration of the cis-acting regulatory elements are shown. The integration of the CECs into the tobacco plastome was designed to occur in the transcriptionally-active spacer region between the *trnI* and *trnA* genes. The wild type (WT) plastome *trnI - trnA* region is shown at the bottom. The expected sizes of *Rsr* II-digested fragments are indicated. Thick black lines represent hybridization sites for the probe used in Southern blot analyses. IEE = Intercistronic Expression Element with the Shine-Dalgarno sequence from the 5' UTR of bacteriophage T7 gene 10 fused to the 3' end; *aadA* = gene encoding aminoglycoside 3' adenylyltransferase; T*psbC* and T*rbcL* = 3' UTRs of *psbC and rbcL* from the white poplar plastome; P*psbA* = promoter and 5’ UTR of tobacco *psbA* gene; mP*rrn* – mutated chloroplast *rrn* operon promoter; *XynA::T* = gene encoding the XynA protein fused at the C-terminal with c-myc and strepII tags. **B**. Transformation with different CECs produced different phenotypes in primary transformants (T_0_). **C**. Southern blot RFLP analysis and confirmation of homoplastomy of T_0_ transformants. Total DNA extracted from 2 independent transformants for each CEC and 1 untransformed plant (WT) was digested with *Rsr* II and analyzed using Southern blotting. All T_0_ transformants displayed a single band of expected size, confirming homoplastomy. **D**. Immunoblot confirmation of XynA expression in plants transformed with different CECs. Lanes 1 through 4 – extracts from CEC1 through CEC4, respectively. Lane 5 – cv. 81V9 WT as negative control. Each lane contains equal amounts of extracted leaf tissue (equivalent to 4.0 mg/lane). Known amounts (ng) of a c-myc-tagged control protein are indicated above the standard curve lanes.

This study was conducted as a part of the Cellulosic Biofuel Network (CBioN, http://www.cellulosic-biofuel.ca) – a Canadian collaborative effort to develop sustainable platforms for biofuels production. The objective of this study was to determine factors that support optimal production of recombinant proteins in transplastomic tobacco as an expression system, with a focus on cellulolytic enzymes. Using a set of regulatory cis-acting elements, we constructed four cassettes expressing the same two foreign genes: *xynA*, encoding a bacterial xylanase from *Clostridium cellulovorans* and the selectable marker gene *aadA* (Figure 1A). We hypothesized that testing levels of accumulation of the foreign proteins produced from different cassettes would determine an ideal configuration, capable of expressing other recombinant proteins at high levels. We also hypothesized that, as recombinant protein bioreactors, some tobacco cultivars could offer different desirable agronomic traits, such as vigorous growth and high biomass yields, which would translate into higher recombinant protein yields.

### Design and construction of the cassettes used in this study

General design and positional configuration of the regulatory cis-elements and foreign genes expressed in all the Chloroplast Expression Cassettes (CECs) was based on previously reported constructs with some modifications (Figure 1A; [11,16,18,33,42,43]). Integration of all the CECs was targeted into the *trnI - trnA* intron in the IR region of the tobacco plastome. No promoters were incorporated into CEC1 and the expression of both the *xynA* and the *aadA* genes from CEC1 relied entirely on read-through transcription from the endogenous promoter of the *rrn* operon (P*rrn*, [44]). CEC2 utilized the read-through transcription for expression of *xynA*, while the *psbA* gene promoter (P*psbA*) along with its 5’ UTR governed the expression of *aadA*. CEC3 was the only cassette designed to contain two strong chloroplast promoters, P*psbA* and mutated P*rrn* (mP*rrn,* see Methods for a description of the mutations; [45]), driving the expression of *aadA* and *xynA*, respectively. CEC4 construct utilized the read-through transcription for expression of *aadA* and the P*psbA*/5’ UTR for expression of *xynA*. The intercistronic expression element (IEE), shown to direct efficient mRNA processing and promote protein expression [28] was incorporated into CEC1 and CEC4 upstream of the *aadA* gene. The 5’ end of the *xynA* reading frame in all the cassettes contained the first 11 codons of the T7*g10* as a translational enhancer [26]; the 3’ end was fused in frame with strepII and c-myc protein tags for detection and purification. The T7*g10* 5’ UTR and downstream box (DB) was located upstream of *xynA* in CEC1, CEC2 and CEC3, while the *psbA* 5’ UTR and T7*g10* DB were used in CEC4. In all the cassettes, the 3’ ends of both *xynA* and *aadA* reading frames were fused to the same heterologous 3’ UTRs from *Populus alba*, T*rbcL* and T*psbC* respectively, required for the stabilization of the mRNA [46,47]. All the described cassettes were cloned into pUC19-based pPT vector [48], designated pCEC1- through -pCEC4-XynA, and propagated in *E. coli*. We observed much lower plasmid DNA yields from bacterial cultures bearing pCECs and 3 – 5 times larger culture volume was required to obtain plasmid DNA yields comparable with the yield of unmodified pUC19 or pPT, indicating possible leaky expression of chloroplast elements in *E. coli* that resulted in probable toxicity and slow growth.

### Generation of transplastomic homoplasmic tobacco plants expressing XynA from different cassettes

Our group previously reported efficient rates of chloroplast transformation achieved in two tobacco cultivars: the low-alkaloid cultivar (cv.) 81V9 and the high-biomass cv. I64 [48]. Cv. 81V9 [49] is used as a model plant in our lab and has been characterized extensively as a platform for transient and nuclear-transformed, stable expression systems [50]. Thus, cv. 81V9 was chosen for the initial selection of the most efficient cassette for transplastomic XynA production. Transplastomic tobacco cv. 81V9 plants were obtained following standard bombardment of leaf tissue with the four cassettes and 3 consecutive rounds of regeneration on selective medium [4,14,16]. High transformation frequencies were observed for all the cassettes, generating usually 10–15 independent transplastomic clones after bombardment of five sterile-grown tobacco leaves.

After the initial PCR-assisted screening confirming foreign DNA insertion (data not shown), the regenerated T_0_ plantlets were rooted on selective medium and grown to a size of 5 – 10 cm before transfer to pots in the greenhouse. Differences in regeneration/rooting timing among the T_0_ plants transformed with different cassettes did not allow for accurate comparison of the growth rate and phenotype, which were observed as roughly similar for CEC1-, CEC2- and CEC4-XynA-transformed plants compared with untransformed wild-type (WT) cv. 81V9 plants. However the CEC3-XynA-transformed plants could be readily distinguished, as they displayed pale-green-to-white leaf color and severely retarded growth (Figure 1B). Two independent T_0_ clones for each cassette were chosen for further experiments; their homoplastomy was confirmed by a Southern blot RFLP analysis that showed stable integration of the foreign DNA into the plastome (Figure 1C).

For the initial analysis of the XynA protein production in T_0_ clones transformed with different cassettes, we sampled young, well-developed leaves of the same size (~30 cm long, ~third-fourth leaf from top), thus minimizing possible developmental variations in XynA expression among the clones and focusing on the cassette effect. Equal amounts of extracted leaf tissue were analyzed by SDS-PAGE and immunoblotting (Figure 1D). The results of this analysis confirmed the expression of XynA from all the cassettes; however, differences in accumulated XynA amounts were observed, suggesting varying expression efficiency from different cassettes. Beside the full-size XynA protein product appearing at ~58 kDa, we also observed two abundant bands of ~27 – 28 kDa in size, detectable with anti-c-myc antibody. These bands correspond to the size of the C-terminal NodB domain of XynA [51].

### *Effects of different* cassettes *on transplastomic plant growth/biomass generation and accumulation of the recombinant proteins*

**Figure 2:**
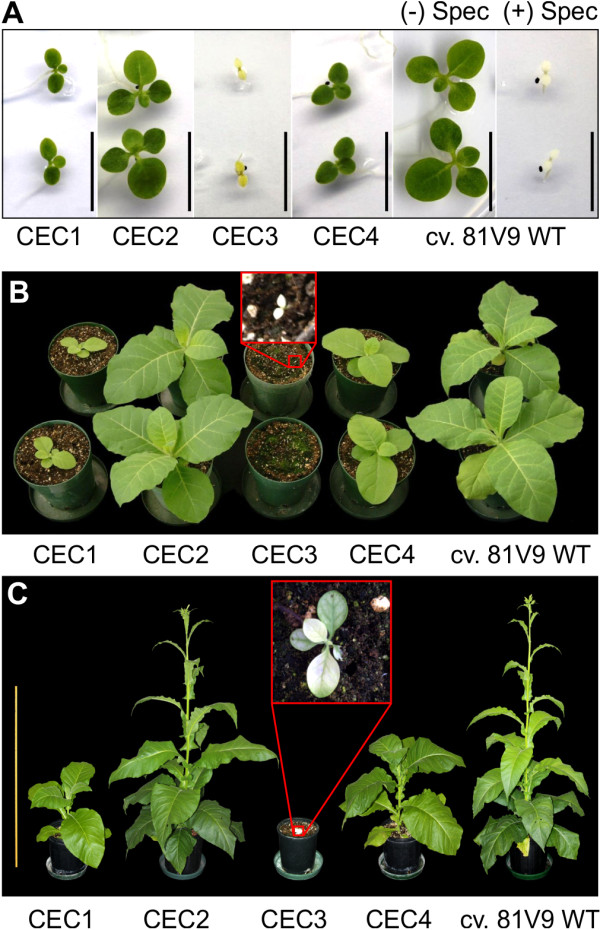
**Synchronized germination and growth of cv. 81V9 T**_**1 **_**plants – phenotype. A**. Phenotypic differences displayed by two-week-old T_1_ transplastomic seedlings (CEC1 through CEC4) germinated on selective medium. Black bar = 1 cm. Untransformed (WT) seedlings germinated on selective (+Spec) and non-selective (−Spec) medium were used as a control. Impact of different cassettes on growth rate of the T_1_ transplastomic plants at 40 days (**B**) and at 80 days (**C**) post-germination is shown in comparison to WT plants.

Sustainability of a plant-based, recombinant protein production system relies on a combination of the plants’ ability to produce adequate biomass yields with sufficient accumulation levels of the recombinant protein. Therefore, even though a cassette can give rise to more recombinant protein per fresh leaf weight than other cassettes, it would not be sustainable to use this cassette if the plant is stunted and gives rise to very little biomass. This apparent paradigm prompted us to compare growth rate, biomass generation, and the accumulation of the XynA protein in T_1_ plants obtained from seeds of self-pollinated T_0_ transformants for each cassette. For that, T_1_ seeds were simultaneously germinated along with WT control seeds. Differences in growth rate and phenotype between the T_1_ seedlings could be observed as early as two weeks post-germination (Figure 2A); those differences appeared much more striking as the T_1_ plants matured (Figure 2B, 2C), and it became clear that CEC3 causes severe growth retardation. Young leaves always looked bleached, and as CEC3 plants grew, older leaves turned light green. Three T_1_ plants for each cassette along with the WT control plants were grown in a greenhouse to maturity (first appearance of flower buds), providing data on the time to reach flowering as well as on fresh leaf weight of a plant at maturity as a parameter for generated biomass (Table 1). CEC3 plants required 307 days to flower, compared with 78 days for WT, and produced less than half the biomass than the WT or any of the other transplastomic genotypes expressing different cassettes (Table 1).

**Table 1:** **Number of days to flowering (DTF) and fresh leaf weight (FLW; kg ± standard error of mean [SEM]; *n *= 3) at mature plant stage in transplastomic low-alkaloids tobacco T**_**1 **_**plants (cv. 81V9) transformed with different CECs expressing XynA**

		**Cassette**			
	**CEC1**	**CEC2**	**CEC3**	**CEC4**	**WT**
DTF	127	80	307	96	78
FLW	0.280^a^±0.01	0.284^a^±0.02	0.127^b^±0.02	0.291^a^±0.01	0.282^a^±0.02
XynA*	4.5±0.33	3.7±0.27	7.5±0.63	18.3±1.39	-

^a^ - Values connected by the same superscript letter are not significantly different (p≤0.05).

* - *Extrapolated* a*mount of intact XynA (mg) produced in one plant.*

**Figure 3:**
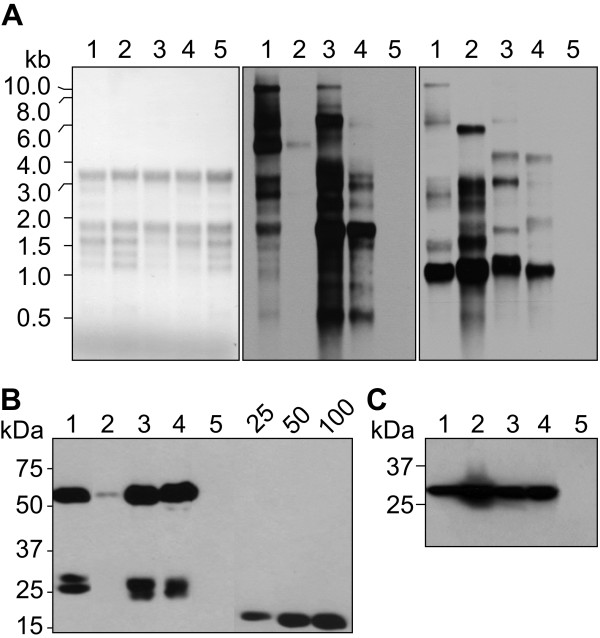
**Analysis of activity of different cassettes introduced into cv. 81V9 T**_**1 **_**plants. A**. Northern blot analysis of *xynA* and *aadA* transcript abundance produced from different CECs. Lanes 1 through 4 represent total plant RNA extracted from T_1_ transplastomic plants for CEC1- through CEC4-XynA, respectively. Lane 5 represents WT RNA. Equal amounts of RNA were loaded in each lane and confirmed by staining (left panel); the blot hybridized to a *xynA*-specific probe (middle panel) and *aadA*-specific probe (right panel) displayed differences in transcript levels and sizes for both genes produced from different CECs. **B**, **C**. Immunoblot of XynA (**B**) and AadA (**C**) protein product produced from different CECs and assessed in the same leaves sampled for the RNA analyses (**A**). Each lane represents 0.4 mg of extracted leaf tissue. Lane 5 - WT leaf extract. Known amounts (in ng) of a c-myc-tagged control protein are indicated above the standard curve lanes (**B**).

To further dissect the effect of the different cassettes on plant phenotype, we compared both *xynA* and *aadA* steady state mRNA and protein levels in the transplastomic plants. For this, leaves of similar size (~30 cm long, ~third-fourth leaf from the top) from T_1_ plants were sampled and analysed (Figure 3A, B, C). Northern hybridizations with gene-specific probes revealed differences in mRNA amounts, as well as transcript sizes, for both *xynA* and *aadA* genes among transplastomic genotypes expressing different cassettes (Figure 3A). The most abundant transcripts for each cassette correlated with the predicted hypothetical sizes of ~4,700 bp (CEC1 and CEC2), ~1,800 bp (CEC3 and CEC4) for *xynA* and ~1,100 bp kb for *aadA*, indicating efficient processing at the heterologous 3’ UTRs. Interestingly, *xynA* transcripts originating from these cassettes revealed considerable quantitative differences, being much more abundant with CEC1 than CEC2 (Figure 3A, middle panel). Given the similarity of the two cassettes (the only difference being that the IEE in CEC1 downstream of *xynA* is substituted with P*psbA* in CEC2), this result was unexpected and the reason for that is not clear. A number of studies utilized constructs with configurations similar to CEC1 [33] or CEC2 [38,39,52], exploiting read-through transcription from the endogenous P*rrn* to obtain high-level recombinant protein accumulation. However, a direct comparison of foreign mRNA levels generated in synchronised plants transformed with such constructs has not been reported. Although unlikely, it is possible that the presence of P*psbA* downstream of the 3’ UTR of *xynA* could affect stability of the UTR’s secondary structure, causing the observed discrepancy in *xynA* mRNA yield between CEC1 and CEC2 through increased degradation by 3’ plastid nucleases. On the other hand, both P*rrn* and P*psbA* are known to incorporate elements recognized by a bacterial-type, multi-subunit plastid-encoded polymerase (PEP), which also includes a nuclear-encoded sigma-factor [53,54], suggesting a possible competition for a functional PEP availability between these promoters, and leading to increased *aadA* transcripts and reduced *xynA* transcripts. Further, CEC2 with swapped positions of *xynA* and *aadA* displayed ~two orders of magnitude lower transformation frequencies compared to other constructs (data not shown), which implies inadequate *aadA* expression (probably at the transcriptional level), necessary to support selective regeneration of transplastomic clones.

CEC3 generated the most *xynA* transcripts among all the cassettes, with the most abundant transcript size being ~1,800 bp, corresponding to a mP*rrn*-generated *xynA* transcript terminated at the T*rbcL* 3’ UTR. The mP*rrn*-driven transcription of the *xynA* gene from CEC3 reached higher levels than CEC1-XynA, which is driven by the endogenous P*rrn*, or of CEC4-XynA, where *xynA* is driven by P*psbA*, whereas these two promoters are considered among the strongest in chloroplast [44,45]. Since each of the three triplet mutations introduced into mP*rrn* was reported to increase transcription ([45]; see Methods section), it is reasonable to suggest that mP*rrn* created in this study is more powerful than native P*rrn*; however, additional quantitative experiments are required to validate this assertion.

XynA and AadA enzymes accumulation from each cassette in T_1_ plants correlated with RNA results (Figure 3B, C). All the cassettes produced AadA at similar levels. CEC2 showed very low *xynA* RNA and protein amounts, while accumulation of XynA expressed from other cassettes reached similar levels. T_0_ plants had displayed a similar effect, albeit not as strong, which could be explained by different ages of T_0_ plants at sampling (compare Figure 3B with Figure 1D). However, although CEC3 appeared to have the highest levels of *xynA* mRNA, at the protein level it produced similar amounts of XynA enzyme as CEC4. Since both transplastomic genotypes transformed with CEC3 and CEC4 displayed similar accumulation levels of both recombinant proteins, the chlorotic stunted phenotype in CEC3-expressing plants is likely due to a disruption of plastid mRNA homeostasis by massively redirecting mRNA synthesis and probably causing reduced transcription of essential genes due to depleted resources of the genetic machinery inside the organelle. To the best of our knowledge this observation that high levels of foreign mRNA accumulation can cause near-lethality is a novel insight into the complexity of transplastomic production of recombinant proteins. This view is different from previous arguments put forward for explaining stunted growth or lethality observed in transplastomic plants, such as foreign protein toxicity and/or a depletion of resources needed for synthesis of essential plastid proteins [25,55-57].

Thus, according to the phenotypic and expression data, although CEC3 produces more transcript and as much protein as CEC4, the plants are severely stunted, and although the developmental pattern and biomass of CEC2 is similar to wild type, it produces very little XynA. As well, CEC1 requires 3 weeks more than CEC4 to reach maturity. Therefore, it appears that CEC4 is the best cassette for XynA production.

**Figure 4:**
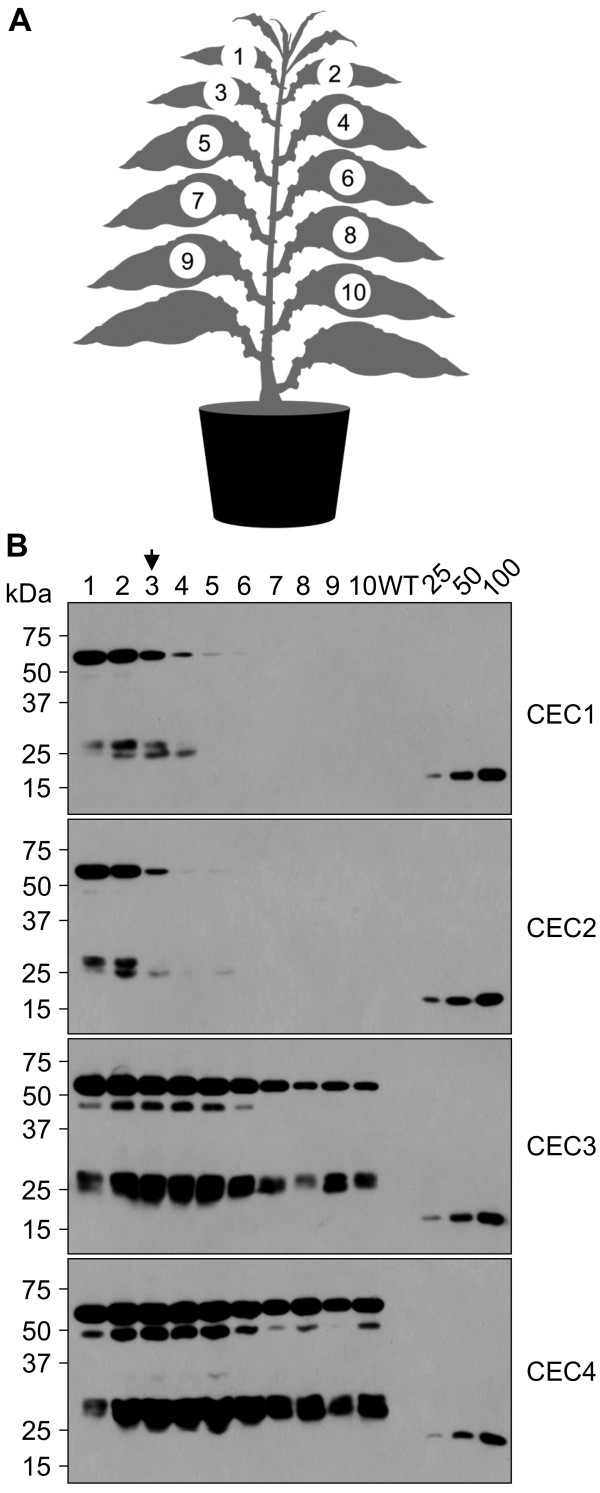
**Spatial accumulation of XynA in cv. 81V9 T**_**1 **_**transplastomic plants transformed with different CECs. A**. Schematic representation of the sampling procedure to obtain spatial accumulation pattern of XynA expressed from different CECs in mature T_1_ transplastomic plants. Samples from ten leaves (top to bottom) were extracted using equal sample weight/buffer volume (w/v) ratio and equal amounts of extracts (each lane represents 4.0 mg of extracted leaf tissue) were analysed by immunoblotting after SDS-PAGE (**B**). The leaf #3 corresponds to the size of a leaf used for initial expression analyses (indicated with an arrow). Untransformed (WT) plants were used as a negative control. Known amounts (in ng) of a c-myc-tagged control protein are indicated above the standard curve lanes.

For the analysis so far conducted, we analyzed *xynA* mRNA and protein production in only one leaf (the third-fourth leaf from the top of the plant). It is possible that younger or older leaves may express XynA differently. To gain insight into the spatial accumulation pattern of XynA in whole mature plants, we sampled 10 leaves (top-to-bottom, Figure 4A) and examined XynA accumulation in equal amounts of extracted leaf tissue by SDS-PAGE and immunoblotting (Figure 4B). According to this analysis, XynA expressed from CEC1 and CEC2 was detected in young leaves only, while accumulation in all leaves was detected in CEC3 and CEC4-transformed plants, with most abundant expression from CEC4. Our observations correlate with the results of Yu et al. [43], who reported that a construct similar to CEC3 produced a cellulase in leaves of all ages, including senescing tissue. Although we did not analyze *xynA* transcript levels in all leaves, these results indicate that a dedicated promoter proximal to the gene of interest (CEC3 and CEC4), rather than endogenous readthrough transcription (CEC1 and CEC2), might lead to better RNA and recombinant protein accumulation in all leaves. The highest accumulation levels of the recombinant intact XynA were observed in younger tissue and were estimated at 0.5% of the leaf total soluble protein (TSP), or ~80 μg/g leaf tissue. We calculated the amount of intact XynA that could be produced in one mature CEC4-transformed cv. 81V9 plant to be ~18.3 mg (Table 1).

### Generation of transplastomic homoplasmic tobacco plants expressing fungal xylanases and effects of T7g10 translational enhancer

**Figure 5:**
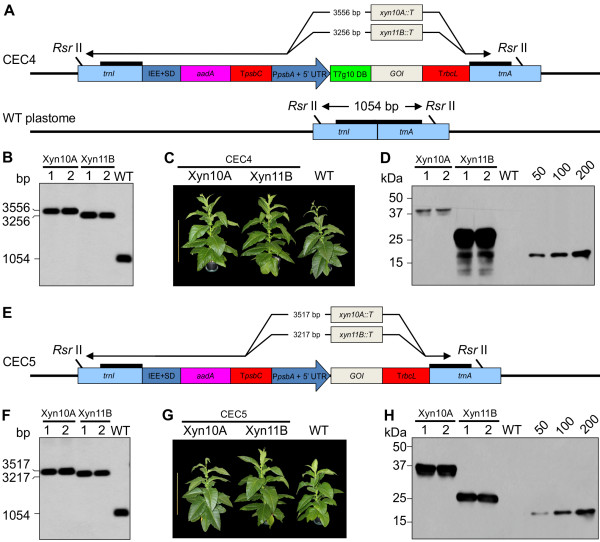
**Constructs for fungal *xyn10A *and *xyn11B *expression; Confirmation of homoplastomy of T**_**0 **_**plants (cv. I64) and effects of T7G10 translational enhancer on accumulation levels of Xyn10A and Xyn11B. A**. CEC4 was used to express fungal xylanases Xyn10A and Xyn11B in high-biomass tobacco cv. I64. The sequences of the *xyn10A* and *xyn11B* genes were cloned into the GOI position of pCEC4.The expected *Rsr* II-generated fragment sizes for Southern blot RFLP analysis are indicated for each construct and for the wild type (WT) plastome. **B**. Southern blot RFLP analysis of cv. I64 T_0_ transplastomic lines transformed with pCEC4-Xyn10A and pCEC4-Xyn11B to confirm homoplastomy, two clones per construct analysed. **C**. Phenotype of T_0_ cv. I64 transplastomic lines is identical to WT plants. **D**. Immunoblot-assisted accumulation analysis for Xyn10A and Xyn11B expressed from CEC4. Two independent primary transformants per construct were examined (lanes 1 and 2 for each protein). Extractions were performed using equal ratio of sample weight/extraction buffer volume (w/v = 1/5). Each lane contains extract equivalent to 4.0 mg of extracted leaf tissue. Untransformed WT extract was used as negative control. Known amounts (ng) of a c-myc-tagged control protein are indicated above the standard curve lanes. **E**. CEC5 construct (identical to CEC4, but lacking the T7*g10* DB element) was used for expression of native forms of Xyn10A and Xyn11B without the T7*g10* N-terminal fusion. **F**. Homoplastomy confirmation was carried out as described above for T_0_ cv. I64 transplastomic lines expressing Xyn10A and Xyn11B from CEC5 (B). **G**. Phenotype of T_0_ cv. I64 transplastomic lines is identical to WT plants. H. Accumulation analysis for Xyn10A and Xyn11B expressed from CEC5 was carried out as described in (D).

Since CEC4 appeared to be the most prolific cassette for XynA production, we sought to further validate it with additional recombinant proteins. For that purpose we used two xylanases from *Aspergillus niger*, Xyn10A and Xyn11B. When tested in a transient, chloroplast-targeted expression system that is being developed in our lab for a rapid evaluation of a protein accumulation potential in chloroplasts, Xyn10A and Xyn11B accumulated to high levels and were found to be non-glycosylated proteins, making them good candidates for transplastomic expression (Conley et al., manuscript in preparation). Two new chloroplast expression constructs were prepared by cloning the original sequences of *xyn10A* and *xyn11B* genes into the GOI position of pCEC4, producing pCEC4-Xyn10A and pCEC4-Xyn11B, respectively (Figure 5A).

With these new constructs we carried out chloroplast transformation of a high-biomass tobacco, cultivar I64, thus testing the performance of the selected CEC4 in a different genetic background. Southern blot RFLP analysis confirmed uniform homoplastomy of the generated cv. I64 T_0_ primary transformants that phenotypically resembled cv. I64 WT plants (Figure 5B, C). Transplastomic T_0_ clones were further examined for the recombinant protein content (Figure 5D). Surprisingly, Xyn10A accumulated only to 13.0 μg/g leaf tissue (0.2% TSP), whereas transplastomic Xyn11B accumulation showed levels reaching 1.3 mg/g fresh leaf tissue (6.0% TSP), consistent with the levels of transiently-expressed, chloroplast-targeted Xyn11B.

Numerous reports found that the N-terminal coding sequence of a protein can strongly affect its accumulation level in chloroplasts [27,36,38,39,58,59]. Chloroplast-produced proteins with N-terminal T7*g10* fusions usually report high levels of expression [27,32,40,41]. Interestingly however, accumulation of neomycin phosphotransferase (NPTII) reporter enzyme, translationally fused with T7*g10* N-terminal portion was significantly improved when the translational enhancer was removed from the expression construct [26]. That result prompted us to scrutinize the impact of the T7*g10* translational enhancer N-terminal fusion on the recombinant protein yields. Two additional constructs, namely pCEC5-Xyn10A and pCEC5-Xyn11B, were prepared by eliminating the DNA fragment encoding T7*g10* translational enhancer from pCEC4-Xyn10A and pCEC4-Xyn11B, respectively (Figure 5E). Primary cv. I64 clones transformed with pCEC5-Xyn10A and pCEC5-Xyn11B displayed uniform homoplastomy and WT-like phenotype (Figure 5F, G). Strikingly, the removal of the T7*g10* translational enhancer greatly increased (more than 16-fold) the accumulation of the Xyn10A, which reached ~0.8 mg/g fresh leaf tissue (3.3% TSP). Contrasting that, the lack of T7*g10* N-terminal fusion was unfavourable for Xyn11B accumulation that decreased by more than two-fold to ~0.4 mg/g (2.5% TSP) (Figure 5D, H). Here we show that T7*g10* N-terminal fusion displayed an opposite effect on accumulation of two different recombinant proteins in chloroplasts, suggesting protein-specific influence for this cis-acting element. Thus, our results imply that a transplastomic approach for expression of recombinant proteins should include testing of combinations of different types of translation control elements for each individual foreign ORF [17].

**Figure 6:**
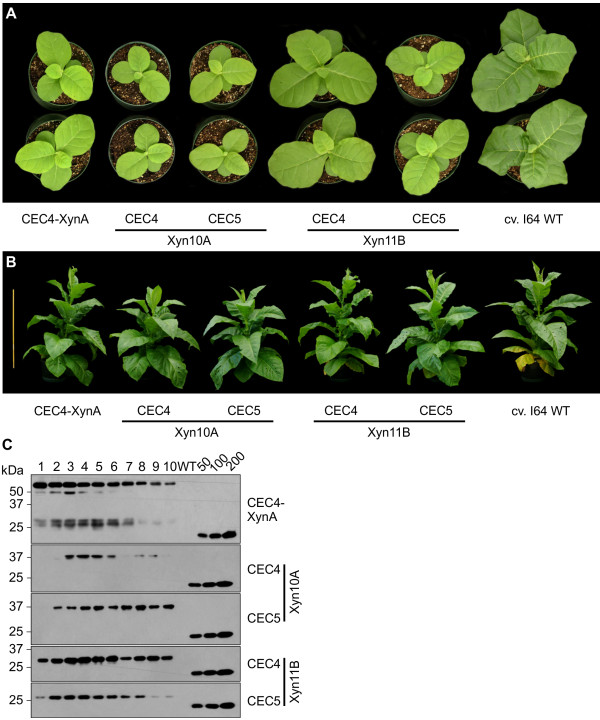
**Tobacco cv. I64 T**_**1 **_**transplastomic plant development and spatial expression patterns of different xylanases. A**. Phenotypes of T_1_ transplastomic cv. I64 plants at 40 days post-germination. A slight developmental delay, which was observed to various extent in the transplastomic lines, was completely compensated during further growth as the plants reached maturity, displaying very similar size and flowering time when compared with WT untransformed plants (**B**, also Table 2). One-meter ruler is pictured on the left for a size reference. **C**. Western blot-assisted assessment of the spatial accumulation profiles of XynA, Xyn10A and Xyn11B in mature cv. I64 plants. Lanes 1 through 10 represent extracts from 10 leaves (top to bottom), each lane represents extract equivalent to 2.5 mg leaf tissue for XynA and Xyn10A expressed from CEC4; for Xyn10A expressed from CEC5 and Xyn11B expressed from CEC4 and CEC5 each lane represents extract equivalent to 0.1 mg leaf tissue. Known amounts (in ng) of a c-myc-tagged control protein are indicated above the standard curve lanes.

We further examined the agronomic performance of the generated transplastomic cv. I64 lines expressing Xyn10A and Xyn11B from CEC4 and CEC5 by simultaneous germination of T_1_ progeny of self-pollinated primary transformants. In addition, observation of a developmental delay of cv. 81V9 line transformed with pCEC4-XynA (Figure 2; Table 1) prompted us to introduce pCEC4-XynA into cv. I64 and to compare T_1_ plants developmental pattern, providing direct comparison of productivity of the two genetic backgrounds as transplastomic expression platforms. Although some differences in growth rate were observed at early stages of development (Figure 6A), all the cv. I64 T_1_ plants were able to grow to a similar size as WT and flower, showing lesser delay than cv. 81V9 T1 plants (Figure 6B; Table 2). Compared with transplastomic cv. 81V9 T_1_ line expressing XynA from CEC4, cv. I64 T_1_ lines, expressing XynA, Xyn10A and Xyn11B from CEC4 and Xyn10A and Xyn11B from CEC5, required somewhat longer time to reach maturity and flower, generating, however, much higher leaf biomass with consistent spatial accumulation of the recombinant proteins, as assessed in 10 leaves of mature plants (Table 1; Table 2; Figure 6C). Examining the best-expressing constructs, we determined that one transplastomic cv. I64 plant, generating ~0.5 kg leaf biomass, could accumulate up to 30.0 mg of XynA, 400.0 mg of Xyn10A and 720.0 mg of Xyn11B (Table 2).

**Table 2:** **Number of days to flowering (DTF) and fresh leaf weight (FLW; kg ± SEM; *n *= 3) at mature plant stage in transplastomic high-biomass tobacco T**_**1 **_**plants (cv. I64) expressing different xylanases from CEC4 and CEC5**

	**CEC4**			**CEC5**		
	**XynA**	**Xyn10A**	**Xyn11B**	**Xyn10A**	**Xyn11B**	**WT**
DTF	109	107	103	104	108	99
FLW	0.551^a^±0.03	0.553^a^±0.02	0.632^a^±0.05	0.551^a^±0.05	0.563^a^±0.03	0.571^a^±0.04
Xylanase*	29.3±3.55	8.8±2.87	719.0±68.37	401.5±37.37	302.1±60.36	-

^a^ - Values connected by the same superscript letter are not significantly different (p≤0.05).

* *Extrapolated* a*mount of recombinant xylanases (mg) produced in one plant*.

### Enzymatic activity of crude plant extracts

**Figure 7:**
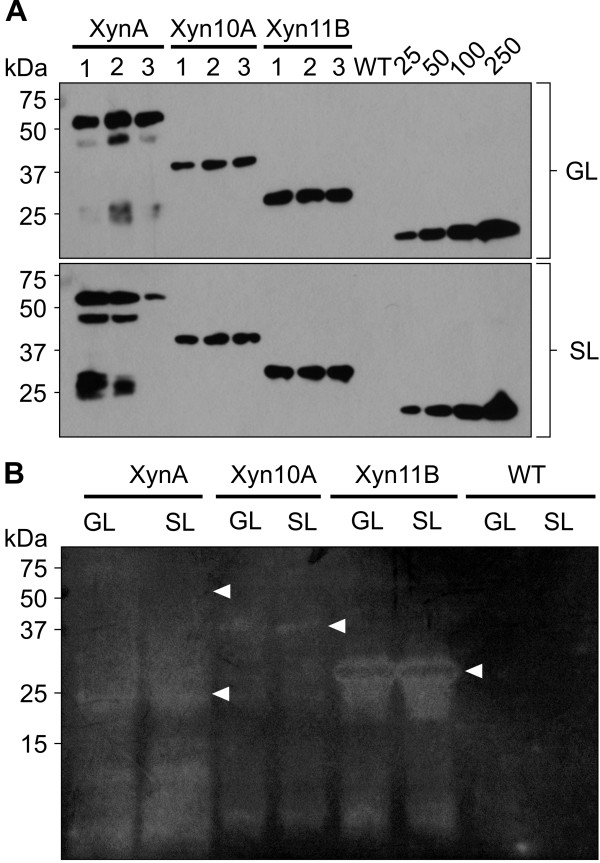
**Determination of enzyme amounts in crude extracts of XynA-, Xyn10A- and Xyn11B-expressing cv. I64 plants measured in mature green leaves (GL) and lower senescing leaves (SL) and a zymogram of the extracts.A**. Three repeated extractions from GL and SL of the cv.I64 lines expressing XynA and Xyn10A from CEC4 and Xyn11B from CEC5 were analysed by western blot for quantification of recombinant proteins content. Each lane of XynA extracts from both GL and SL represents 2.5 mg of extracted leaf tissue; for Xyn10A and Xyn11B each lane represents 0.05 mg and 0.25 mg of extracted leaf tissue for GL and SL extracts, respectively. WT – extract of untransformed WT plants used as a negative control. Known amounts (in ng) of a c-myc-tagged control protein are indicated above the standard curve lanes. **B**. Zymogram of GL and SL extracts (combined of three repeats) for XynA, Xyn10A and Xyn11B, resolved on a 12% SDS-PAGE gel containing 0.1% (w/v) xylan. Each lane contains equivalent of 2.5 mg of extracted leaf tissue. Equal amount of extracts from WT leaves were used as a negative control. Arrows indicate expected sizes of the protein bands detected on western blots.

Efficient and sustainable conversion of lignocellulosic biomass to ethanol requires an abundant and inexpensive supply of active cell-wall-degrading enzymes. Transplastomic plants expressing different cellulases can potentially provide a cost-effective strategy for the production of cellulosic ethanol [33,60,61]. Indeed, the cost of enzymatic hydrolysis of cellulosic biomass could be further reduced by the use of crude plant protein extracts, making expensive procedures for enzyme purification unnecessary [19,60]. Therefore, we tested enzymatic xylanolytic activity of crude plant extracts from transplastomic cv. I64 lines best-expressing XynA, Xyn10A and Xyn11B by incubation with Birch wood xylan as a substrate, monitoring the release of reducing sugars in xylose equivalent [62,63]. The T7*g10* N-terminal fusion had no effect on enzymatic activity of the chloroplast-produced xylanases (data not shown), thus, only the most productive cv. I64 lines expressing XynA and Xyn10A from CEC4 and Xyn11B from CEC5 were analyzed. Further, a recent study reported reduced activity of several chloroplast-expressed cellulases in aged (lower) leaves of transplastomic tobacco plants [60]. This prompted us to examine xylanase activity in extracts from mature green leaves (GL) and old leaves undergoing senescence (SL). The amounts of recombinant xylanases were determined in the same extracts, allowing calculations of xylan to xylose conversion efficiency, as well as enzyme activity expressed as μmol xylose generated per μg enzyme (Figure 7A; Table 3).

**Table 3:** Xylanolytic activity of crude plant extracts from mature green leaves (GL) and senescing leaves (SL) of cv. I64 transplastomic lines expressing XynA, Xyn10A and Xyn11B

	**Enzyme Crude Extract**							
	**XynA**		**Xyn10A**		**Xyn11B**		**WT control**	
Leaf developmental stage	GL	SL	GL	SL	GL	SL	GL	SL
Enzyme in reaction (μg)	1.7±0.15	1.5±0.5	45.9±4.3	11.3±1.1	83.2±1.9	16.8±0.3	-	-
Generated Xylose (μMole)	146.1±4.3	245.4±7.1	169.6±2.9	319.33±5.5	159.1±2.4	209.67±4.11	0.7±0.4	8.5±2.1
% Conversion*	21.8	36.6	25.3	47.7	23.7	31.3	0.1	1.3
Generated Xylose (μMole)/enzyme (μg)	85.9	163.6	3.7	28.3	1.9	12.5	-	-

Crude extracts (400 μl, representing 40 mg of extracted leaf tissue) were added to 10 ml of 1% (w/v) xylan and incubated 24 hours at 40°C with shaking. Enzyme in reaction and generated xylose values represent means of 3 different extraction repeats ± SEM.

* Initial amount of xylan in reaction is 670 μMole (0.1 g; >90% xylose residues).

The results showed that equivalent amounts of crude extracts containing different amounts of XynA, Xyn10A and Xyn11B, generated 21.8 to 47.7% conversion of xylan. Xyn10A produced the highest conversion efficiency, although it accumulated to lower levels than Xyn11B. Thus, Xyn10A seems to be more catalytically active in crude extracts than Xyn11B. Although the conversion efficiency of XynA appeared lower than that of Xyn10A, when corrected for amount of enzyme present in the reaction, XynA was vastly superior to both fungal enzymes in its ability to hydrolyze xylan and produce xylose, (Table 3). XynA is a major xylanase subunit of the cellulosome from *Clostridium cellulovorans* and its high enzymatic activity could be attributed to the synergy in action of its two catalytic domains: the N-terminal catalytic domain and C-terminal NodB, with xylanase and acetyl xylan esterase activities, respectively [51]. The domains are separated by a small dockerin domain that “docks” the protein into the cellulosome by its interaction with a receptor domain – cohesin on the cellulosomal structural protein scaffoldin CbpA [64]. That dockerin domain is probably an easy target for plastid proteases – on protein blots from XynA extracts we observed abundant bands corresponding to molecular sizes of the NodB domain with or without the dockerin (~27 – 28 kDa), suggesting it has two protease cleavage sites (Figure 1D, 3B, 4B, 6C). Yet, the separated domains retained their catalytic activity, which was observed in a zymogram (Figure 7B). The intact XynA band could not be distinguished in the zymogram; instead, a smeary clearing appeared in lanes loaded with XynA extracts, indicating presence of partially degraded XynA protein, undetectable with anti-c-myc antibody, and possible failure of XynA to refold in its intact form after denaturing SDS-PAGE. Although the activity assay indicated that Xyn10A is more catalytically active than Xyn11B, the zymogram displayed the clearest band for Xyn11B, correlating with its higher accumulation. This observation implies that crude extracts may have different effects on stability/activity of the fungal xylanases accumulated in chloroplasts, while SDS-PAGE conditions could provide physical segregation from probable inhibiting and/or degrading agents present in crude plant extracts, allowing the separated enzymes to “work” on their substrate in the “protected environment” of the gel matrix.

We observed higher xylanase activity in extracts from SL for all three recombinant enzymes, whereas the amount of the fungal Xyn10A and Xyn11B diminished ~4-fold, compared to GL tissue (Table 3). This result was unexpected and may be due to induction of plant endogenous cellulases/hemicellulases in the SL tissue, which is supported by the elevated levels of reducing sugars obtained in the control reactions with SL extracts from WT leaves. Yet, this increase cannot account for the massive increase in reducing sugars content observed in the reactions with extracts from SL tissue of the transplastomic lines. A more probable explanation to the observed phenomenon is the presence of an inhibitory factor, which acts in the GL tissue and is depleted from the SL tissue. Indeed, several reports described occurrence of specific endogenous protein inhibitors of xylanases in different plant species, including tobacco [65-68]. Although additional studies are required to confirm that the foreign xylanases accumulated in chloroplasts are catalytically subdued in crude extracts by a specific inhibitor(s) found in fresh leaves, it is reasonable to suggest that identification of such inhibitors in tobacco and targeted knock-out of their genes through genetic manipulation could lead to creation of cultivars”tailored” for expression of xylanases belonging to a particular type or family, which would be highly catalytically active in plant crude extracts.

## Conclusions

In this study we optimized the transplastomic production of recombinant xylanases in tobacco for a potential application in bioethanol industry. The initial optimization steps focused on selection of the most efficient chloroplast expression cassette, which combined recombinant protein expression/leaf biomass generation maxima as productivity parameters. Using the selected cassette we demonstrated that different genetic backgrounds, chosen as a platform for transplastomic expression in tobacco, allow additional optimization for the production process of recombinant enzymes.

## Methods

### Chloroplast transformation cassettes construction

The sequences of the cis-regulatory elements were chemically-synthesized and assembled into the designed cassettes by a series of restriction/ligation reactions (Figure 1A). A *xynA* clone (AF435978; [51]) was a generous gift from Dr. Yutaka Tamaru, Mie University, Japan. *Xyn10A* and *xyn11B* sequences were provided by Dr. Adrian Tsang, Concordia University, Canada. Primers used for amplification/cloning of the GOIs into cassettes are listed in Additional file 1: Table S1A in Additional Materials section.

In all the cassettes constructed, the 3' ends of the *xynA* and the *aadA* genes were fused to the 3' UTR regions of *rbcL* (T*rbcL*) and *psbC* (T*psbC*) plastid genes from White Poplar (*Populus alba*, chloroplast genome NC008235; nucleotides 56790-to-57022 and 34875-to-35052, respectively).

Three triplet nucleotide mutations (“-58 – -56” ATG to TAC; “-22 – -20” AGG to TCC and “-5 – -7” CTC to AGA mutation; [45]) were introduced into the sequence of the chloroplast *rrn* operon promoter (*Nicotiana tabacum* chloroplast genome NC001879, nucleotides 102565-to-102648) producing mutated P*rrn* (mP*rrn*) in order to increase the transcription, also reducing the homology to the endogenous P*rrn*, thus reducing the chance of deleterious homologous recombination between these sequences.

Integration of the cassettes into the tobacco plastome was designed to occur between the *trnI (tRNA-Ile)* and *trnA (tRNA-Ala)* genes. For that, the transformation cassettes were introduced into the *Nsi* I site in the *trnI-trnA* intron of the pPT vector, described in [48], thus creating Chloroplast Expression Cassette vectors (pCECs), designated as pCEC1–C4 (Figure 1A). The sequences of the GOIs were cut with *Sap* I (*Nhe* I for CEC5) and *Not* I and introduced into all the pCECs by direct cloning into the corresponding restriction sites.

### Generation of transplastomic plants and confirmation of homoplasmy

Transplastomic tobacco plants (cv. 81V9 and I-64) were obtained by the biolistic method [4,14,16]. After 3 rounds of regeneration on selective medium containing 500 μg/mL spectinomycin, homoplasmy of all the clones was confirmed by Southern blot analysis (Figure 1C; Figure 4B, F). Three μg of plant total DNA, extracted from the transplastomic clones as well as from untransformed WT plants (Qiagen DNeasy Plant Mini kit, Qiagen, GmbH), were completely digested with *Rsr* II enzyme, electrophoresed on 0.8% agarose gel and transferred onto Hybond-N+ membrane (Amersham Biosciences, UK). A DIG-labelled probe was amplified with primers Probe-F: 5’-caccacggctcctctcttctcg-3’ and Probe-R: 5’-ttcctacggggtggagatgatgg-3’ using PCR DIG Probe Synthesis kit (Roche Diagnostics, GmbH) and pPT as the template, according to the manufacturer’s protocol. Hybridization of the probe was carried out at 50°C over-night. Three stringency washes (100 mL of 2XSSC + 0.1% SDS at RT – twice; 100 mL of 0.5XSSC + 0.1% SDS at 68°C – three times) were performed, followed by 30 min blocking at 42°C and 30 min of antibody binding. Detection was carried out with AGFA CURIX ULTRA EJUTK film with different exposures.

### RNA extraction and northern blot analyses

Total plant RNA was isolated using the RNeasy Plant Mini Kit (QIAGEN Sciences, Maryland, USA) according to the manufacturer’s instructions. For each RNA sample, 2 μg were electrophoretically separated on a denaturing, 1.2% agarose gel. Following capillary transfer of the RNA to a nylon membrane (Roche Diagnostics, GmbH), the membrane was submerged in a reversible staining solution (0.02% Methylene Blue, 0.3 M sodium acetate, pH 5.5) for 5 minutes. The membrane was then washed in 1× SSC until the background had cleared such that the consistency of the transfer and the quality of the RNA could be visualized (Figure 3A, left panel). Subsequently, the damp membrane was put into the pre-hybridization solution prepared using DIG Easy Hyb Granules (Roche) according to the manufacturer’s instructions. The blot was probed at 50°C with a DNA fragment of Xylanase A that had been DIG-labelled using the PCR DIG Probe Synthesis Kit (Roche Diagnostics, GmbH). Stringency washes were employed (2×5 minutes at room temperature with 2× SSC, 0.1% SDS; 1×15 minutes at 68°C with 0.5× SSC, 0.1% SDS; 1×15 minutes at 68°C with 0.1× SSC, 0.1% SDS) before blocking with Blocking Reagent (Roche Diagnostics, GmbH) and detection with Anti-DIG Fab fragments and CSPD (Roche Diagnostics, GmbH) as described by the manufacturer. The blot was subsequently exposed to X-ray film for various times to visualize the hybridized bands.

### Recombinant protein extraction, quantification and functional enzyme analyses

For extraction of total soluble proteins from leaf tissue ~0.05 g samples frozen in liquid N_2_ were homogenized in Tissuelyser (Qiagen, GmbH) for 2 minutes in 2-mL Eppendorff tubes with 3 silica beads (Biospec, USA), then either ~250 μl or ~500 μl of Extraction Buffer (EB; 50 mM Na-Acetate, 15 mM CaCl_2_, pH 4.9) were added to the tubes to obtain 1/5 or 1/10 sample weight/extraction buffer volume (w/v) ratios, vortexed for 1 minute and centrifuged for 10 minutes at 14000 X g, 4°C. The supernatant was used as crude extract for quantification of the expressed recombinant proteins as well as for enzymatic activity analyses.

Immunoblot analyses were performed to assess levels of recombinant protein expression. For that, crude extracts were resolved on 12% SDS-PAGE gels, transferred onto a nitrocellulose membrane by semi-dry electroblotting (Biorad, USA). The blots were blocked over night at 4°C in 5% skimmed milk in Tris-buffered Saline (TBS, pH=7.5) and subsequently probed with a primary antibody, either anti-c-myc (Genscript, USA) or anti-AadA (Agrisera, UK), diluted 1:5000 in 0.5% skimmed milk-TBS for 1 hour; the horse radish peroxidase-conjugated goat anti-mouse IgG (secondary antibodies, Biorad, USA) was diluted 1:5000 in 0.5% skimmed milk-TBS and incubated with the blots for 1 hour. The recombinant proteins accumulated in transplastomic leaf tissue were quantified from immunoblots by densitometry with TotalLab TL100 software (Nonlinear Inc., Durham, USA) using intensity analysis of specific bands, where known amounts of a c-myc-tagged CBD protein were used as reference.

Enzymatic hydrolysis of birchwood xylan (Sigma, USA) by crude extracts (w/v = 1/10) of XynA-, Xyn10A- and Xyn11B-expressing plants was carried out in 15 ml tubes. Extract from WT plants was used as negative control. Crude extracts were prepared in EB, 400 μl of each extract representing 40 mg of extracted leaf tissue were mixed with 10 ml of 1% (w/v) xylan as a substrate, diluted in EB. Reactions were set as triplicates for each extraction at 40°C for 24 hours with agitation, and then placed on ice for 30 min. Subsequently tubes were centrifuged and the supernatant (40 μl) was mixed with 70 μl of Dinitrosalicylic Acid (DNS) reagent [62], boiled for 5 min and examined in a spectrophotometer (Bio-Rad) for reducing sugar content [62,63].

A zymogram of the xylanolytic activity of crude extracts from XynA-, Xyn10A- and Xyn11B-expressing plants (equivalent of 2.5 mg of extracted leaf tissue) was obtained on 12% SDS-PAGE gel containing 0.1% (w/v) birchwood xylan (Sigma, USA), followed by 3×30 min washes in 100 ml of EB to eliminate SDS and renature the resolved proteins. Subsequently, the gel was incubated in 100 ml of EB at 40°C for 4 hours, stained with 0.1% Congo Red and de-stained in 1M NaCl.

### Statistical analyses

Extractions of total soluble proteins for each experiment were repeated at least 3 times. From those, samples for protein and enzymatic analyses were taken. At least 6 technical repeats were analyzed for each extraction. Collected data were used to express the mean value of a parameter ± standard error of the mean (SEM). Values were analyzed using JMP software (SAS Institute, Cary, USA).

## Abbreviations

aadA: Aminoglycoside adenylyltransferase gene; xynA: Gene encoding XynA xylanase fom *C. cellulovorans*; xyn10A: Gene encoding Xyn10A xylanase fom *A. niger*; xyn11B: Gene encoding Xyn11B xylanase fom *A. niger*; CEC: Chloroplast expression cassette; GOI: Gene of interest; GL: Mature green leaf tissue; SL: Senescing leaf tissue; TBS: Tris-buffered saline; TSP: Total soluble protein; trnI: *trn-Isoleucine* gene; trnA: *trn-Alanine* gene; IR region: Inverted Repeat region

## Competing interests

The authors declare that they have no competing interests.

## Authors’ contributions

IK designed the constructs, produced transplastomic plants and analyzed expression and activity of the enzymes, AK carried out northern and western blot experiments, SL and EP provided ideas and feedback, RM conceived of the study, and participated in its design and coordination and helped to draft the manuscript with IK. All authors read and approved the final manuscript.

## Supplementary Material

Additional file 1: Table S1A Primers used for amplification/cloning. Click here for file
