# Supplementary material for: Optimization of transplastomic production of hemicellulases in tobacco: effects of expression cassette configuration and tobacco cultivar used as production platform on recombinant protein yields
Source: Biotechnol Biofuels. 2013 May 3;6:65. doi: 10.1186/1754-6834-6-65 (PMC3655837; doi:10.1186/1754-6834-6-65)
Supplement: Additional file 1: Table S1A — Primers used for amplification/cloning. [file 1754-6834-6-65-S1.docx]

Table A1. Primers used for amplification/cloning of the GOIs

*The genes sequences were PCR-amplified, incorporating Sap I and Nhe I to the 5’ ends of the GOIs and* *Not I restriction sites (underlined) and the sequences encoding c-myc- and strepII-tags (TAGS) fused to the 3' ends for subsequent cloning and to facilitate purification/detection with anti-c-myc antibody.*

| Gene | Primers |
| --- | --- |
| *xynA* | XynA-F:  5’-caTCGCTCTTCTATGGCAACAAAAACGATCACCTCAAATGAGACTGG-3’ |
|  | XynA-TAGS-R:  5’-CAAGCGGCCGCTTATTTTTCGAACTGCGGGTGAGACCACAGATCTTCTTCAGAGATCAGTTTTTGTTCGAATGCACCATTTAACATTGTACCAAGTTG-3’ |
| *xyn10A* | Xyn10A-F:  5’-TAGAGCTCTTCTATGgctagcCCCATTGAACCCCGTCAGG-3’ |
|  | Xyn10A-TAGS-R:  5’-CAAGCGGCCGCTTATTTTTCGAACTGCGGGTGAGACCACAGATCTTCTTCAGAGATCAGTTTTTGTTCGAGAGCATTTGCGATAGCAGTGTATG-3’ |
| *Xyn11B* | Xyn11B-F:  5’-TAGAGCTCTTCTATGgctagcGTTCCCCACGACTCTGTGG-3’ |
|  | Xyn11B-TAGS-R:  5’-CAAGCGGCCGCTTATTTTTCGAACTGCGGGTGAGACCACAGATCTTCTTCAGAGATCAGTTTTTGTTCCTGAACAGTGATGGACGAAGATCCA-3’ |
